# Biotype dynamics of the common house mosquito *Culex pipiens* in the anthropogenic environment in winter: a citizen science approach

**DOI:** 10.1038/s41598-026-55124-5

**Published:** 2026-06-13

**Authors:** Rody Blom, Arnold J. H. van Vliet, Jolijn van Gils, Jet S. Griep, Manon A. H. Oudejans, Constantianus J. M. Koenraadt

**Affiliations:** 1 Laboratory of Entomology, Plant Sciences Group, Wageningen University & Research, Wageningen, The Netherlands; 2 Earth Systems and Global Change Group, Wageningen University & Research, Wageningen, The Netherlands

**Keywords:** Ecology, Ecology, Zoology

## Abstract

The two biotypes of the mosquito species *Culex pipiens s.s.* (biotype *pipiens* and biotype *molestus*) differ in overwintering strategy. Biotype *pipiens* enters diapause, whereas biotype *molestus* remains active year-round. In the Netherlands, flooded crawlspaces may provide sheltered development sites for biotype *molestus* in winter. We therefore investigated the effects of human population density and crawlspace flooding on the presence of *Cx. pipiens s.s.* biotypes and their hybrids using citizen-submitted mosquito samples via the platform ‘Muggenradar.’ Mosquitoes were categorized by population density and crawlspace condition. In addition, biotypes and feeding patterns were identified using molecular analyses. Descriptive analyses indicated that biotype *molestus* was more abundant in areas with high population density (24.1% at ’low’ versus 58.6% at ‘high’), while biotype *pipiens* dominated in areas of low population density (19.5% at ‘high’ versus 63.3% at ‘low’). Similarly, we found an association of biotype *molestus* with flooded crawlspaces (68.4% of specimens). However, these patterns were not fully supported by a multivariate analysis. Blood meal analyses revealed that nearly all biotype *molestus* (98.2%) and hybrid mosquitoes (100%) fed on humans. Surprisingly, six blood-engorged biotype *pipiens* were collected. We conclude that anthropogenic environments may influence the occurrence of *Cx. pipiens* s.s. biotypes and hybrids in winter.

## Introduction

In recent years, several mosquito-borne viruses, such as Usutu (USUV), West Nile virus (WNV) and Sindbis virus (SINV) have emerged in North- and North-Western Europe^1–4^. In the Netherlands, these viruses were discovered for the first time in the last decade^5–7^. They circulate within a mosquito-bird sylvatic cycle, and *Culex pipiens* s.s. (sensu stricto) is considered an important vector of these viruses in North-Western Europe. *Culex pipiens* s.s. has proven to be a competent vector of these viruses in laboratory studies^8–15^. Additionally, all three viruses have been isolated from wild *Cx. pipiens* s.s. populations^16–21^. The sibling species *Culex torrentium* has shown to be a competent vector of USUV and WNV in laboratory studies, but their vectorial capacity is considered low due to their low abundance in the Netherlands^22–25^. In addition to being a competent vector of several viruses, *Cx. pipiens* s.s. is a (potential) vector of other pathogens of medical and veterinary concern, such as avian *Plasmodium*^26^.

*Culex pipiens* s.s. is characterized by its omnipresence, high abundance, and complex genetic makeup, which is reflected in strong phenotypical differences between populations^27,28^. Two morphologically identical but behaviourally distinct biotypes of *Cx. pipiens s.s* can be discriminated: *Cx. pipiens* biotype *pipiens* and *Cx. pipiens* biotype *molestus.* Both biotypes can occur sympatrically and produce hybrids, which are presumed to display intermediate behaviour^24,29,30^. Given their morphological indistinguishability, the two biotypes can only be discriminated using molecular markers associated with the biotypes, such as CQ11, COI and *ace*-2^31^. Both biotypes and their hybrids are presumed to play different roles in maintenance of the sylvatic cycle and spill-over of WNV and USUV to dead-end hosts of WNV and USUV, due to differences in their vector competence, behaviour and ecology^9,14,23,31,32^. Biotype *pipiens* is anautogenous, meaning that females require a blood meal prior to producing their first clutch of eggs. Contrastingly*,* biotype *molestus* is autogenous, meaning females do not require a blood meal to produce the first clutch of eggs. Based on laboratory studies, both biotypes are presumed to differ in their feeding behaviour, as biotype *pipiens* is considered to be ornithophilic (preference for birds), whereas biotype *molestus* is considered to be mammophilic or anthropophilic (preference for mammals or humans, respectively)^33,34^. Interestingly, in the field, both biotypes and their hybrids display plasticity in their feeding behaviour^22,29,30,35–37^. As such, the presumed feeding preferences are not always reflected by the feeding behaviour observed in the field.

One of the most striking behavioural differences between both biotypes is their overwintering strategy^24,38,39^. All insects, and thus also mosquitoes, are ectothermic, and therefore many insect species require an overwintering strategy to be able to withstand unfavourable climatic conditions during winter at northern latitudes^40,41^. In autumn, shortened daylight (below 11.5 h) and a decrease in temperature (below 20 °C) prompts larvae of biotype *pipiens* to initiate diapause as adults^38,39,42^. Diapause is a pre-programmed physiological state that allows the insect to overcome adverse conditions during winter. It is characterized by low metabolic activity, cessation of ovariole development, and termination of blood feeding^43–45^. Biotype *pipiens* generally overwinters in cool environments with stable temperatures and high relative humidity^46^. In contrast, biotype *molestus* is homodynamic, meaning it is unable to enter diapause and, as a consequence, it needs to continue feeding on blood in winter^24^. However, the winter activity of biotype *molestus* is understudied, and it is unclear on which hosts they feed in winter.

The two biotypes also differ in their mating behaviour. Biotype *pipiens* requires large, open spaces to mate (eurygamy), whereas biotype *molestus* can mate in small, confined spaces (stenogamy)^47^. Because of this, the latter is commonly associated with subterranean anthropogenic habitats, such as (flooded) basements, sewers, tunnels, and rainwater catch basins^48^. Given the fact that biotype *molestus* remains active in winter, we hypothesize that these subterranean habitats are required to maintain their lifecycle. In the Netherlands, many houses have crawlspaces, a small (usually < 1 m in height) open area underneath the house. High ground-water levels and/or ground subsidence causes crawlspaces to flood, providing a potential larval habitat for mosquitoes. Anecdotal evidence suggests that biotype *molestus* may utilize flooded crawlspaces as an oviposition site in winter (personal observation). This could indicate that the lifecycle of this biotype is maintained because of the continued availability of oviposition sites in flooded crawlspaces in winter. Furthermore, we expect that dry crawlspaces are a suitable overwintering site for diapausing biotype *pipiens,* given that these spaces are relatively cool and without any disturbances. Although crawlspaces seem well closed off to the outside environment, they are also often equipped with ventilation grids that may provide mosquitoes access to shelter or a potential breeding site.

Even though *Cx. pipiens* s.s. is a ubiquitous species associated with anthropogenic habitats, there are differences in habitat choice between the two biotypes. The presence and abundance of biotype *molestus* is more strongly associated with subterranean habitats in urban areas, in contrast to biotype *pipiens*, which is more associated with aboveground habitats^27^. However, empirical evidence for these habitat associations is ambiguous and inconclusive, because of other environmental gradients (e.g. latitude). Moreover, most studies investigating these patterns have been conducted during summer, and it remains unclear whether the associations observed in summer are representative of overwintering populations^23,35,36^.

Mosquito research in domestic environments is challenging due to low accessibility of private property. However, rather than entering and investigating these private properties one by one, data can also be collected using a citizen science approach. In such a case, citizens would contribute by reporting observations and/or specimens to a central location. Earlier studies have indeed shown that indoor collections of mosquitoes can be performed with the help of citizens^49–52^ and provide valuable insights in the winter ecology of *Cx. pipiens* s.s. in domestic environments^24^.

Using a citizen-science approach, we aimed to assess the question whether and how human population density affects the relative occurrence of both *Cx. pipiens* s.s. biotypes and their hybrids. We then investigated whether the presence of (flooded) crawlspace affects the relative occurrence of *Cx. pipiens* s.s. biotypes. In addition, we also assessed host-feeding of both biotypes and hybrids and identified biotype-specific host-feeding patterns based on blood meal analyses of mosquitoes submitted to our project. To collect the data, we initiated a call within our mosquito citizen science project ‘Muggenradar’^24^. Through Muggenradar, Dutch citizens were requested to fill in a questionnaire and send mosquitoes collected indoors to our laboratory for subsequent analyses.

## Results

**Fig. 1:**
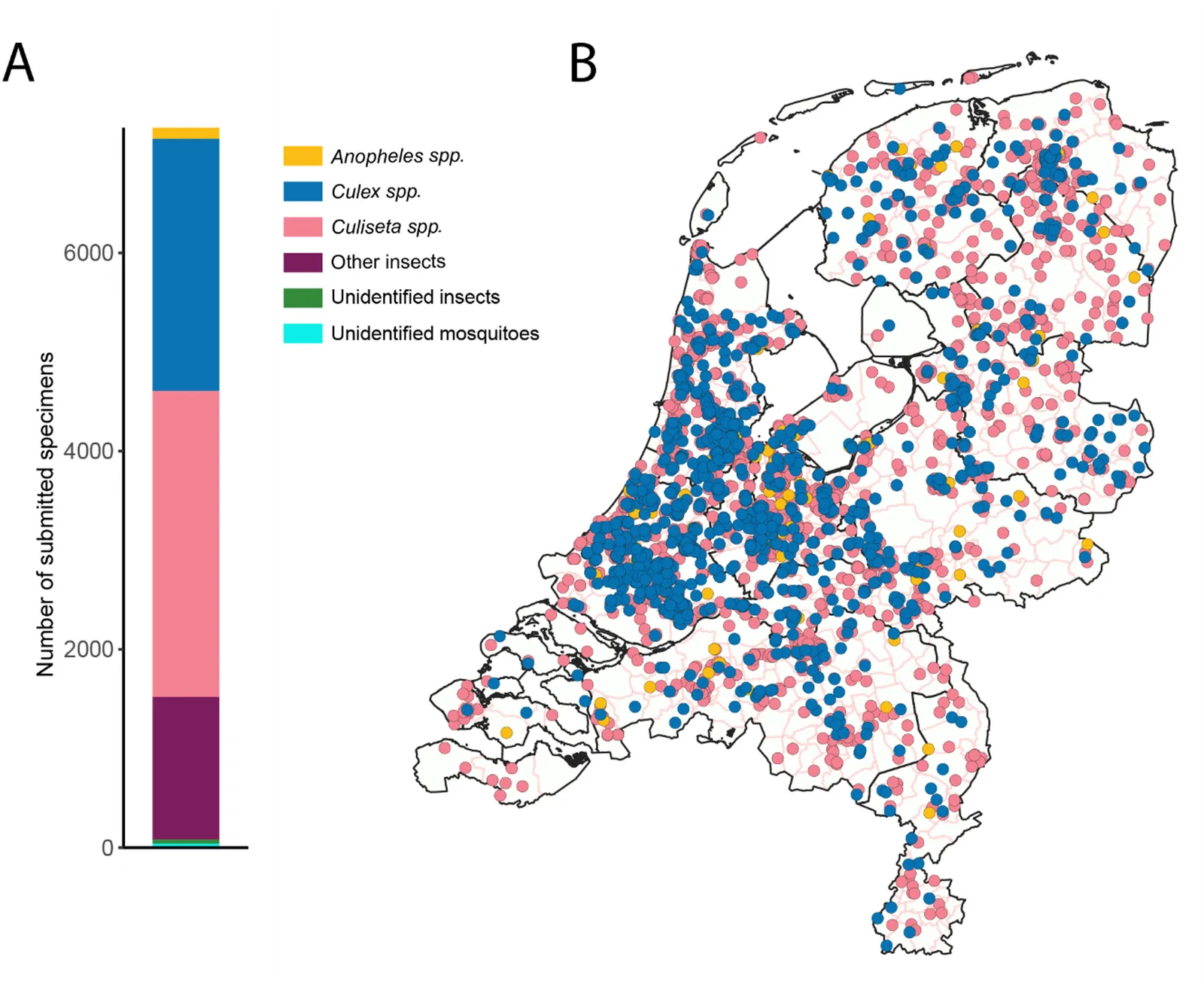
(**A**) Stacked bar chart displaying the results of morphological identification of the 7264 specimens submitted via the Muggenradar citizen science platform. (**B**) Map highlighting the locations from which mosquito samples were submitted; same colour coding as for panel A. Muggenradar submissions containing other insects are not depicted on the map. The map was created by the authors, using QGIS software (version 3.22 ‘Białowieża’^53^;). Administrative boundary data were obtained from Statistics Netherlands (CBS): ‘CBS Gebiedsindelingen 2016–2025’ (https://www.cbs.nl/nl-nl/dossier/nederland-regionaal/geografische-data/cbs-gebiedsindelingen).

Between February 16 and February 26, 2021, a total of 7943 submissions were registered with the online form. This resulted in 5365 submitted samples, which contained 7264 insects. Of these, 5744 (78%) insects were identified as mosquitoes. Other submitted insects belonged to Anisopodidae and Trichoceridae, but we did not conduct any further species identification. Subsequent morphological identification of mosquitoes resulted in 3088 *Culiseta spp.* (53.7%), 2544 *Culex spp.* (44.3%), and 112 *Anopheles spp.* (2.0%) (Fig. 1). The level of participation differed strongly between the provinces. The highest participation level was observed in the province of Utrecht (4.8 submissions per 10,000 inhabitants), whereas the lowest level of participation was in the province of Limburg (0.8 submission per 10,000 inhabitants) (Table S2). Questionnaire responses provided additional context on where participants had observed mosquitoes indoors during the study period. Mosquitoes were most frequently reported from living rooms (51.7%) and bedrooms (48.9%), followed by kitchens (20.7%). Attics (4.0%) and basements (1.0%) were reported less frequently. Other locations (13.9%) primarily included bathrooms, restrooms, and study rooms. Because respondents could report multiple indoor locations, percentages do not sum to 100%. Regarding the number of mosquitoes observed indoors, the majority of respondents reported 1–5 mosquitoes (61.2%), while 6–10 mosquitoes (11.3%) and more than 10 mosquitoes (16.7%) were reported less frequently. Additionally, 10.8% of respondents indicated that they were unable to estimate the number of mosquitoes observed indoors. From the 5365 submitted samples, 5019 were used for further analyses, based on the criteria (associated submission form and geolocation) mentioned earlier.

### Human population density

**Fig. 2:**
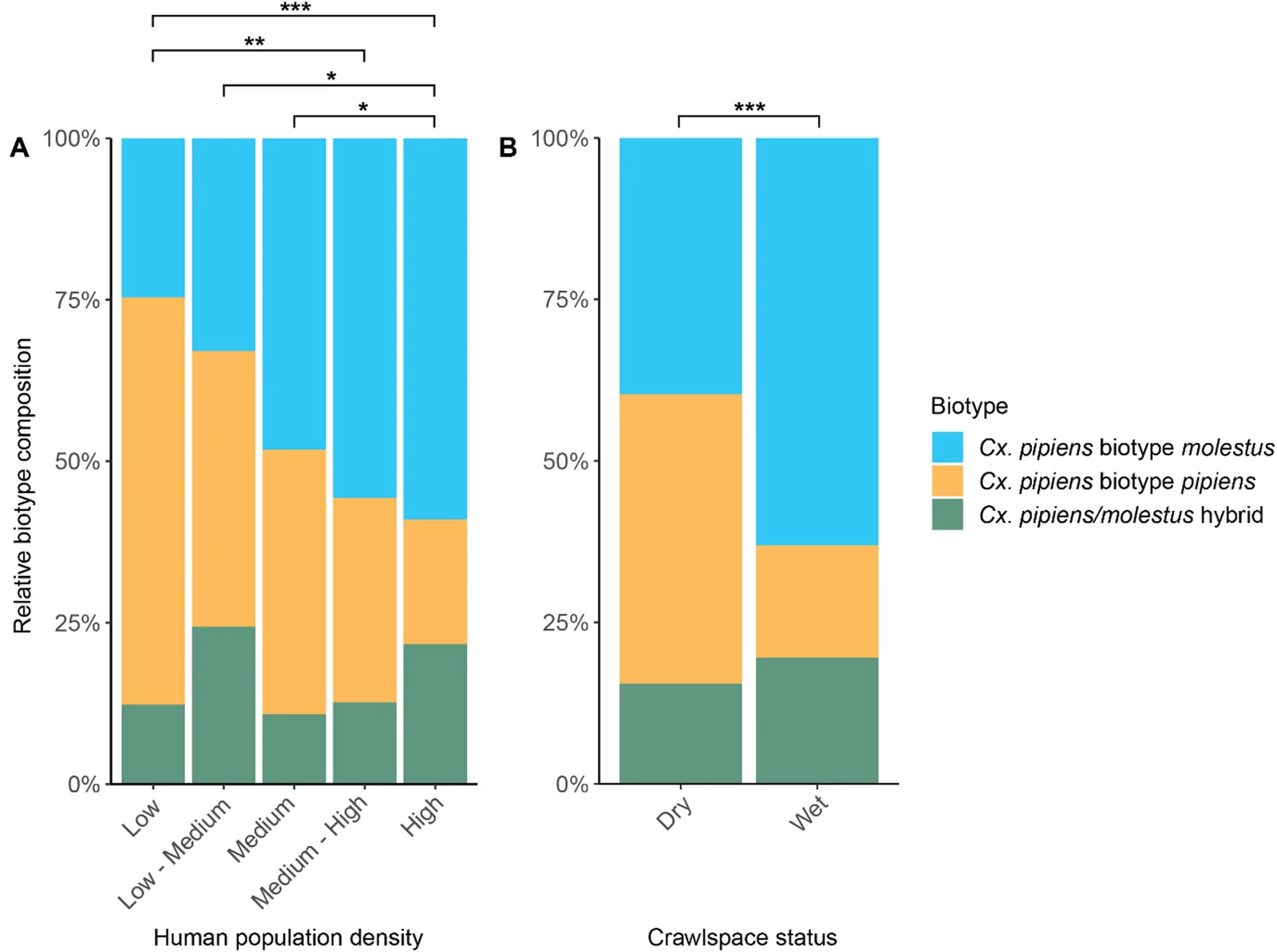
(**A**) Relative biotype composition of *Cx. pipiens* s.s. biotypes and hybrids submitted from residences located in one of five different human population density levels. Human population density levels were based on postal code density, with the following categories: low (< 500 postal codes/km^2^), low-medium (500–1000 postal codes/km^2^), medium (1000–1500 postal codes/ km^2^), medium–high (1500–2500 postal codes/km^2^), and high (> 2500 postal codes/km^2^). (**B**) Relative composition of *Cx. pipiens* s.s. mosquito biotypes and *pipiens/molestus* hybrids submitted from residences with a flooded crawlspace or dry crawlspace. Both bar charts represent biotype proportions based on the respective subsets. Significance of pairwise comparisons is highlighted with accolades. Significance codes: < 0.001 ‘***’, < 0.01 ‘**’, < 0.05 ‘*’.

From a subset of 440 mosquitoes, biotype ratios differed significantly between human population density levels (no. of postal codes/km^2^, χ^2^ = 44.32, *df* = 8, *p* < 0.001). Overall, the relative abundance of biotype *pipiens* was lower at high human population density levels (19.5% at ‘high’ versus 63.3% at ‘low’), and vice versa for biotype *molestus* (24.1% at ’low’ versus 58.6% at ‘high’). Pairwise comparison of biotype abundances between human population density levels showed significant differences between several groups (Fig. 2A, Table S3). To assess whether these patterns remained when also accounting for crawlspace status, a multinomial regression was performed. In this model, human population density levels were significantly associated with the relative occurrence of biotype *pipiens*, with significant effects observed at low-medium (*p* < 0.05) and high (*p* < 0.001) human population densities. From the 440 analysed samples, 23 did not yield a result for biotype *pipiens*, biotype *molestus* or *pipiens/molestus* hybrids, nor for *Cx. torrentium*.

### Crawlspaces

Out of 5,019 submissions used in our analyses, 3648 (72.7%) residences had a crawlspace. Of these, 579 (15.9%) were (partially) filled with water. A relatively high proportion of flooded crawlspaces can be observed in the coastal provinces compared to the inland provinces (Figure S1). In coastal provinces, 20.0% of the submissions originated from a residence with a flooded crawlspace. In inland provinces, this was only 10.8% (Figure S1). The highest percentage of flooded crawlspaces was in the province of Zeeland (30.4%) which is in strong contrast to the province of Limburg (4.3%) (Table S2). From a subset of 115 tested samples, a statistically significant difference in *Cx. pipiens* s.s. biotype ratios between residences with dry and wet crawlspaces was observed (χ^2^ = 13.63, *df* = 2, *p* < 0.001). A higher proportion of biotype *molestus* specimens was submitted from residences with a flooded crawlspace (68.4%), compared to those with a dry crawlspace (37.1%). Contrastingly, the percentage of biotype *pipiens* submitted from residences with a dry crawlspace (41.9%) was higher compared to those submitted from residences with a flooded crawlspace (14.0%)(Fig. 2B). However, the multinomial analysis did not indicate a significant effect of crawlspace status on biotype composition (*p* > 0.05) when also including the human population density effect in the model. For 5 out of 115 samples, the molecular species identification did not result in the identification of biotype *pipiens*, biotype *molestus*, *pipiens/molestus* hybrids, or *Cx. torrentium*.

### Blood meal identification

In total, 250 of the *Culex spp.* (10.5%), 123 of the *Culiseta spp.* (4.3%), and 7 of the *Anopheles spp.* (7.1%) contained visible traces of blood. The 250 blood-fed *Culex spp.* were selected for subsequent blood-host identification. In total, 245 blood-engorged *Cx. pipiens/torrentium* mosquitoes were analysed by Real-Time PCR to identify the biotype and by PCR and Sanger sequencing to identify the blood meal origin. Remaining samples were lost in processing. Biotype analysis resulted in 27 hybrids (11.0%), 200 *molestus* biotype (81.6%), and six *pipiens* biotype specimens (2.4%). Analysis of twelve mosquitoes did not result in a positive signal for either of the biotypes (4.9%). None of the samples tested positive for *Cx. torrentium*.

**Fig. 3:**
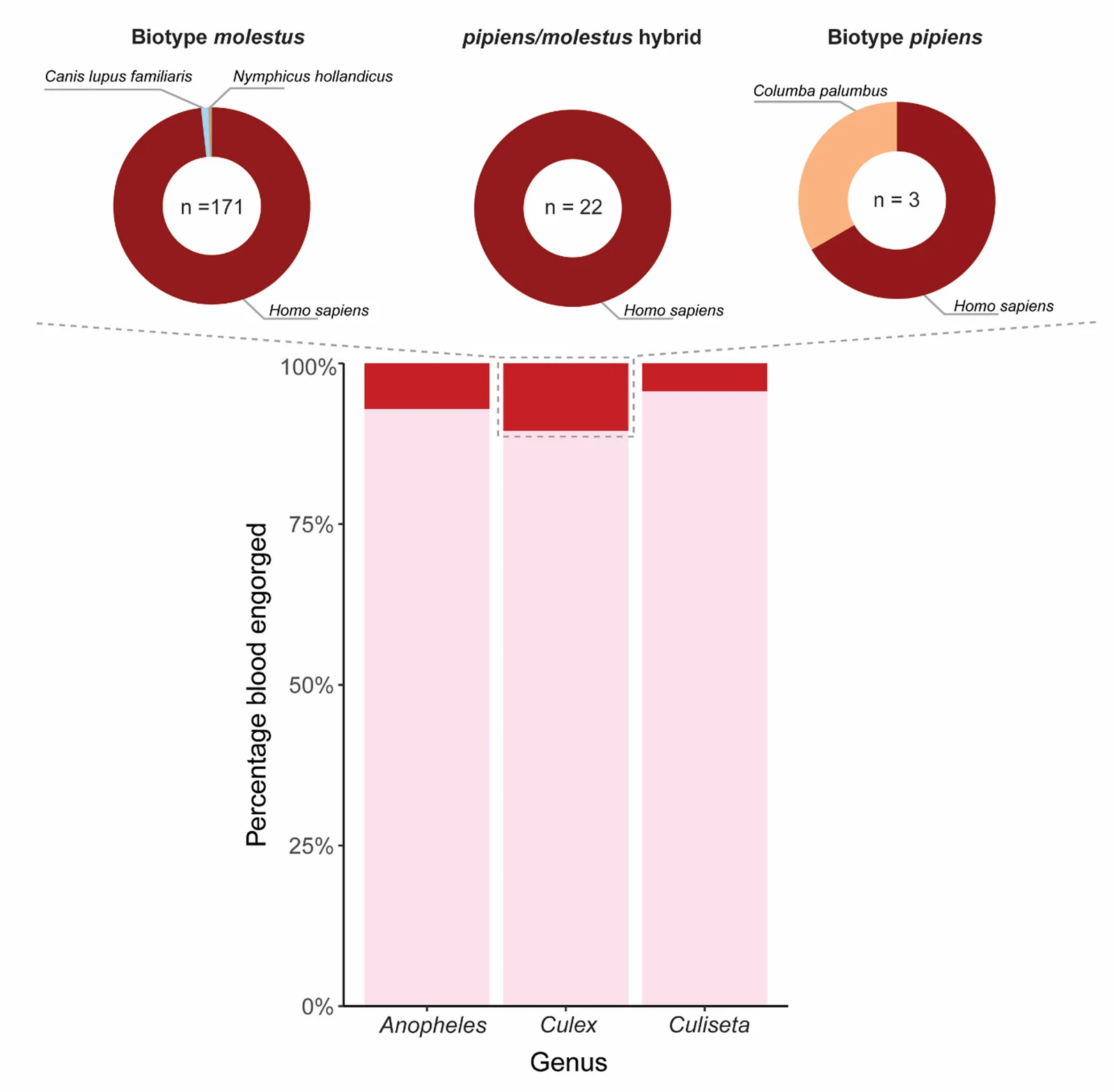
Stacked bar chart displaying the percentages of blood-engorged specimens per mosquito genus. Blood-feeding patterns of *Cx*. *pipiens* s.s. biotypes and *pipiens/molestus* hybrids collected indoors in winter are highlighted in the donut charts. Blood host identities of *Culiseta* and *Anopheles* mosquitoes were not determined.

Out of 5019 submissions, 2398 (47.8%) were sent from residences with pets. From the 159 submissions that contained one or more blood-engorged *Cx. pipiens* s.s. mosquitoes, 68 (42.8%) participants indicated they live with one or more pets. From these, 201 mosquitoes (82.0%) had taken a blood meal from a human (*Homo sapiens*) host. In contrast, only six mosquitoes (2.4%) had taken a blood meal from an animal host. These hosts included one chicken (*Gallus gallus*), one cockatiel (*Nymphicus hollandicus*), one wood pigeon (*Columba palumbus*), one cow (*Bos taurus*), and two dogs (*Canis lupus familiaris*). For 38 mosquitoes (biotype *pipiens*: 3, biotype *molestus*: 23, *pipiens/molestus* hybrids: 4, unsuccessful biotype identification: 8), the blood meal host origin could not be identified, because the PCR was unsuccessful with both primer pairs, or because the sequencing results did not match with a sequence in the NCBI BLAST database. The wood pigeon was bitten by a *Cx. pipiens* biotype *pipiens*, whereas the dogs and the cockatiel were bitten by a *Cx. pipiens* biotype *molestus* (Fig. 3, Table S4). RT-PCR did not yield a biotype for the mosquitoes that had fed on the chicken and the cow. Humans were bitten by *molestus*, *pipiens,* and *pipiens/molestus* hybrids.

## Discussion

This study elucidates the ecology of *Cx. pipiens* s.s. biotypes and hybrids in indoor environments during winter. We found that the relative proportion of biotype *molestus* was higher from residences situated in urbanized areas than those from residences situated in rural areas. Furthermore, the presence of flooded crawlspaces was positively associated with the relative abundance of biotype *molestus.* However, this latter pattern was not consistently supported when analyzed using a multinomial regression. Host-feeding analyses showed that most mosquitoes had fed on humans, as expected. Surprisingly, six blood-engorged biotype *pipiens*-mosquitoes were submitted.

### Relative biotype composition

We observed an association between human population density and the proportion of *Cx. pipiens* s.s. biotypes and hybrids. Higher population densities coincided with a higher proportion of biotype *molestus* mosquitoes. These results are in line with the classical view of biotype *molestus* being the ‘urban’ biotype of the two. In this study, we randomly selected from five different human population density categories, and we thus did not explicitly account for the spatial distribution of the submitted samples. Therefore, submissions may have been unevenly distributed over the country, with local clustering around high human population areas (Fig. 1).

Despite these limitations, the observed association with high human population density (and therefore urban environments) is in line with the classification of biotype *molestus* as an ‘urban’ biotype. It remains unclear whether biotype *molestus* occupies other (natural) habitats in the absence of suitable anthropogenic habitats. It should be noted, however, that our study was performed in winter and may therefore differ from the relative biotype distributions in summer. In our study, the percentage of hybrids ranged between 10.5% and 21.8%. In other studies, hybridization rates between 1.7% and 31% have been reported^23,24,48,54–59^. The majority of those studies were performed in summer, with exception of the study from Vogels et al.^24^. In latter study, hybridization rates ranged between 10.3% and 14.6%, which are similar to the lower end of the percentages found in our study. Interestingly, in our study, relatively high proportions of hybrids were found at postal code densities of 500–1000 and 2000–2500 addresses/km^2^. Based on literature, we assumed that hybridization will primarily occur in areas with high relative abundance of biotype *molestus*, which may move to aboveground habitats where they hybridize with biotype *pipiens*^54^. However, because of the relatively high proportion of hybrids in the low-medium human population density level, this hypothesis could not be confirmed.

Additionally, local environmental conditions, such as crawlspace flooding, may further influence biotype composition. Descriptive analyses (Pearson’s chi-square) indicated a higher proportion of biotype molestus in flooded crawlspaces, compared to dry crawlspaces. However, the multinomial analysis did not support this finding. These observations are in line with our hypothesis that flooded crawlspaces may be used as larval habitat by biotype *molestus*. We did not investigate whether flooded crawlspaces were actually occupied by immature stages of biotype *molestus*, although anecdotal evidence suggests that this is the case. To verify the utilization of flooded crawlspaces by biotype *molestus* as a larval habitat, we suggest conducting follow-up studies in which flooded crawlspaces are actively sampled, either via larval dipping and/or aquatic environmental DNA (eDNA) sampling. eDNA sampling has proven to be a valuable tool in the detection of premature stages of mosquitoes in aquatic habitats^60–63^. In theory, citizen science and eDNA studies can be combined, as the general public can be requested to take water samples and send them to the laboratory. In earlier ecological studies, citizen science and eDNA have been combined, and have proven to be useful^64,65^. However, the combination of eDNA and citizen science has not yet been used for research on vectors. Interestingly, we observed that the coastal provinces of the Netherlands had a relatively higher percentage of flooded crawlspaces compared to the provinces inland, which most likely is a result of coastal provinces being at a lower altitude than inland provinces. However, some of the coastal provinces also contain many highly urbanized settlements (figure s2), which, as we show in our study, also strongly shapes the relative abundance of *Cx. pipiens* s.s. biotypes and hybrids.

### *Cx. pipiens* s.s. phenology

**Fig. 4:**
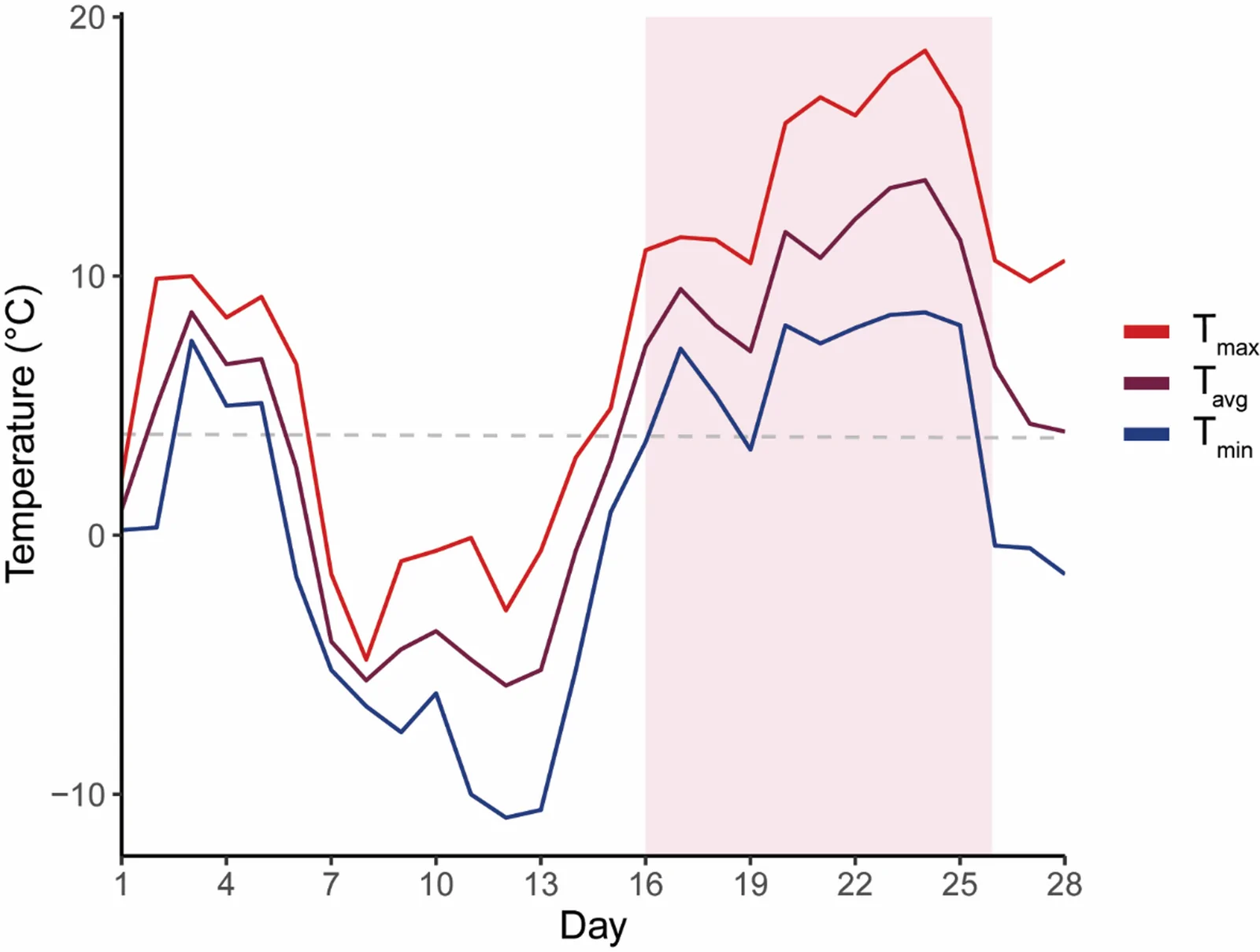
Minimum (T_min_), maximum (T_max_), and average (T_avg_) temperature in February 2021, recorded at one field station (De Bilt) in the Netherlands^67^. After the 12th of February, temperatures increased, up to a maximum of 18.7 °C on the 24th of February. The Muggenradar sampling period lasted from the 16th until the 26th of February, as highlighted in pink. The dashed grey line represents the average outdoor temperature (4.3 °C) over the entire month of February.

The current paradigm is that biotype *pipiens* enters diapause before winter, a period during which they do not actively feed on hosts^38^. Surprisingly, we observed three winter-active biotype *pipiens* specimens with identifiable host origin in mid-February, which were all collected from residences in (peri-)urban environments. In an earlier study performed in California (USA), blood-engorged and gravid *Cx. pipiens* s.s. (biotype unspecified) were collected throughout the entire winter period^66^. Furthermore, average winter temperatures in their study lie well above average winter temperatures in the Netherlands, which could explain the winter activity as observed in their study. At the time of our study, temperatures were unusually high for the time of the year, reaching peak temperatures of 18.7 °C on the 24th of February (Fig. 4)^67^. The unusually high temperatures are a likely explanation for the activity of biotype *pipiens*, biotype *molestus*, *pipiens/molestus* hybrids, *Culiseta spp.*, and *Anopheles spp*. in our study. Global climate change is expected to lead to considerably warmer and shorter winters in North-Western Europe^68,69^. It is expected that such changes in climate causes shifts in the overall phenology, and particularly the diapause dynamics of biotype *pipiens*^70^. As a result of shorter and warmer winters, shifts in the timing of the induction and termination of *Cx. pipiens* diapause may occur^42,71^. Furthermore, changes in winter climate induce shifts in the phenology of other organisms on which *Cx. pipiens* s.s. is dependent (e.g. the flowering period of plants), which – in turn—may influence the diapause period of biotype *pipiens.* However, the possible effects that shifts in the diapause period of biotype *pipiens* may have on arbovirus overwintering and transmission remain unclear. Another explanation for our observation of winter-active biotype *pipiens* could be that the genetic distinction between diapausing and winter-active mosquitoes is not as clear as previously assumed. Consequently, this could lead to some biotype *pipiens* mosquitoes being classified as the *pipiens* biotype using the genetic marker (CQ11) that we used in our study, whereas they exhibit behaviour that is classically associated with biotype *molestus* and vice versa. Furthermore, for some specimens we were unable to identify the biotype, which can be due to initial morphological misidentification. The genetic marker we used in our study is generally considered to be robust for the distinction between biotype *pipiens*, biotype *molestus* and *pipiens/molestus* hybrids. Nonetheless, it is worthwhile to further investigate the population genetics of diapausing- and winter active mosquitoes.

### Host-feeding patterns

Within all three genera, we found a relatively high proportion of blood-engorged mosquitoes in our study. Usually, the proportion of blood-engorged mosquitoes collected in field studies in summer is low. Blood-engorged mosquitoes are difficult to collect using CO_2_-based trapping, but the use of resting boxes and manual aspiration from resting sites may prove to be effective^72,73^. Our study demonstrates that a citizen science setup is effective in collecting blood-engorged mosquitoes that can subsequently be used to identify mosquito host-feeding patterns. It should be noted, however, that the vast majority of *Cx. pipiens s.s.* (regardless of biotype) blood meals had a human origin. This was expected, as citizen science approaches for the collection of blood-engorged specimens is biased towards mosquitoes that have fed on human hosts in the domestic environment. Nonetheless, six of the blood-engorged mosquitoes in our study had fed on an animal host. Two mosquitoes had fed on a cow (*B. taurus*) and chicken (*G. gallus*), two species which are kept domestically, but generally not indoors. The mosquito that had fed on a cow was submitted from a small rural village in the south of the Netherlands, which has several dairy farms in the vicinity. Possibly, the mosquito had fed at a dairy farm, after which it moved indoors after feeding. However, the nearest dairy farm is located at approximately 1 km from the location of collection, and the flight distance of blood-engorged mosquitoes does often not exceed 150 m^74,75^. The mosquito that had fed on a chicken was collected from a peri-urban location, with no nearby poultry farms. Therefore, it seems likely that this mosquito had fed on a backyard chicken. One of the blood-engorged biotype *pipiens* fed on a wood pigeon (*C. palumbus*), a bird species which is generally not kept as pet. In this particular case, it is likely that feeding occurred outdoors, after which the mosquito went indoors to digest the blood meal.

The consequences of blood-feeding by *Cx. pipiens* s.s. during winter for arbovirus maintenance remain unclear. Despite the fact that both biotype *molestus* and biotype *pipiens* had host-fed in winter in our study and are competent vectors of several arboviruses, it is unlikely that they encounter and feed on a viremic reservoir host in winter, and especially indoors^10–12,14^. Furthermore, it is important to note that the feeding activity of biotype *pipiens* in our study occurred after a period of exceptionally high temperatures, potentially causing this biotype to terminate diapause and reinitiate host-feeding. Such periods of exceptionally high temperatures are unusual, but are expected to increase in the future^76^. Therefore, we expect that arbovirus maintenance via winter-active mosquitoes is unlikely. However, possibly, prior to winter, biotype *molestus* mosquitoes become infected via the uptake of an infectious blood meal, after which they transmit the pathogen to their progeny via vertical transmission. There is no evidence of vertical transmission of WNV nor USUV in biotype *molestus* mosquitoes, but vertical transmission of SINV in biotype *molestus* has been demonstrated before^77^. Mosquitoes in diapause are still considered to be the primary arbovirus overwintering route. It is known that diapausing biotype *pipiens* and *Cx. torrentium* mosquitoes can act as winter reservoir for USUV, WNV, and SINV, albeit at very low infection rates^16,19,78–81^. However, projected future increases in winter temperature due to global climate change may increase the number of infected mosquitoes that survive throughout winter. This, in turn, may affect virus prevalence in spring and summer, although this aspect remains understudied. Furthermore, the role of other overwintering routes (i.e. persistence in avian- or non-avian hosts) remains unclear. In light of recent emergence of WNV and annual cases of USUV in the Netherlands^5,6^, it is important to continue research on the different potential overwintering routes and environmental conditions (e.g. temperature) of these arboviruses and the role of vectors and hosts therein.

### Other mosquito species

Interestingly, a large number of *Culiseta* mosquitoes were submitted. In this study, we focused on *Cx. pipiens* s.s., and therefore did not identify *Culiseta* mosquitoes to the species level. However, we noticed that the vast majority were *Culiseta annulata*. Adults of *Cs. annulata* can be found in hibernacula together with *Cx. pipiens/torrentium, Culex territans*, and *Anopheles maculipennis* s.l., where they overwinter^82,83^. Although it is known that, in general, *Cs. annulata* is relatively cold-tolerant compared to other mosquito species, no research on the genetic basis for winter activity and diapause in this species has been performed^84^. Therefore, it is unclear whether multiple forms, or biotypes, may exist, as is the case with *Cx. pipiens* s.s. In summer, *Cx. pipiens/torrentium*, or more specifically, *Cx. pipiens* s.s. is usually one of the most commonly collected mosquito species in CO_2_-baited mosquito traps in the Netherlands, instead of *Cs. annulata*^85,86^. Therefore, it was interesting to observe that the number of *Culiseta* mosquitoes submitted to our laboratory was higher than *Cx. pipiens* s.s. This observation highlights the potential of citizen science approaches to generate large numbers of specimens, which provides valuable opportunities to study *Culiseta* mosquitoes in greater depth. Future studies which include identification and genetic analyses on the species level would help determine whether this pattern reflects seasonal differences, sampling bias, or underlying ecological or physiological adaptations of *Culiseta* mosquitoes. Such analyses were beyond the scope of our study, but future work could explore the ecology and overwintering strategies of *Culiseta* mosquitoes.

### Conclusion

Human population density and crawlspace status (dry or flooded) were associated with differences in the relative occurrence of *Cx. pipiens* s.s. biotypes and hybrids in our descriptive analyses. The positive association of biotype *molestus* with higher human population density and flooded crawlspaces highlights the impacts of anthropogenic activities on arbovirus vector communities. Furthermore, we show that both biotype *pipiens*, biotype *molestus*, and *pipiens/molestus* hybrids can actively blood-feed in winter, at least during periods when temperatures are exceptionally high. Further research should focus on the implications of anthropogenic activities and climate change on *Cx. pipiens* s.s. communities in winter and winter activity combined with arbovirus transmission. Involving the public as part of a citizen science program has proven to be successful in addressing questions on mosquito ecology that are difficult to tackle with routine (trap-based) mosquito sampling approaches.

## Methods

### Mosquito collection and processing

From 16 to 26 February 2021, the Dutch general public was requested to participate in the citizen science project ‘Muggenradar’. Participants were recruited through a press release on NatureToday.com, which generated media attention and increased engagement. In brief, people were asked to collect one or multiple mosquitoes indoors, and send these to the Laboratory of Entomology, Wageningen University & Research. To maintain identification characteristics, participants were asked to kill the mosquitoes carefully. Via the Muggenradar website (www.muggenradar.nl), participants could access a submission form, which was used to register their collected specimens. Every entry was linked to a unique six-digit code, and postal codes were provided by the participants. All submission forms contained a questionnaire with additional questions (Table S1). After arriving at our laboratory, envelopes were stored at -20 °C until further processing. In some cases, participants submitted samples without submitting a form. In most of those cases, the postal code was provided on the envelope. All methods performed in this study were in accordance with the Dutch privacy regulations^87^. All participants provided informed consent to use the information they provided in the online form and the biological material they submitted (insects) in our analyses. Samples without a submitted form and postal code were discarded. In some cases, participants submitted multiple insects (including non-mosquitoes). Subsequently, mosquitoes were identified to the genus level using the morphological identification key by Becker et al.^88^ and stored in Eppendorf tubes at -20 °C until further processing. Adult female *Cx. pipiens* s.s. are nearly indistinguishable from the sibling species *Cx. torrentium*. Therefore, prior to molecular identification, *Cx. pipiens* s.s. and *Cx. torrentium* were grouped together as *Cx. pipiens/torrentium*. Samples that had an associated filled-out online submission form and could be geolocated were considered for further sample selection of the different analyses. All methods and protocols (including data collection and processing) were performed in accordance with the Netherlands Code of Conduct for Research Integrity. Methods and protocols were reviewed by the Research Ethics Committee of Wageningen University and Research (WUR-REC) and deemed minimal risk.

### Human population density and crawlspace flooding

To determine the effect of population density on the relative occurrence of *Cx. pipiens* s.s. biotypes, the population density was determined of each location from which a sample was sent. This was done using information retrieved from the ‘Kerncijfers wijken en buurten’-database developed by Statistics Netherlands (Centraal Bureau voor de Statistiek (CBS)) and the postal codes given by respondents in the questionnaires^89^. In the CBS database, all municipalities are categorized in one of five categories based on the number of postal codes per km^2^, which we used as a proxy for human population density. For the purpose of our study, we used these five predefined categories: low (< 500 postal codes/km^2^), low-medium (500–1000 postal codes/km^2^), medium (1000–1500 postal codes/km^2^), medium–high (1500–2500 postal codes/km^2^), and high (> 2500 postal codes/km^2^). A random selection of up to 90 *Culex* mosquitoes was made per category, except for the ‘low’ human population density category. Only 80 *Culex* mosquitoes were selected in this category, as only 80 suitable samples were submitted from that category. No stratification by sample density or geographic location was applied. The subset of samples was used to assess the relationship between human population density and *Cx. pipiens* s.s. biotype composition.

In addition, the effects of crawlspace flooding on *Cx. pipiens s.s.* biotype and *pipiens/molestus* hybrid occurrence were assessed. For this purpose, participants were asked whether their residences have a crawlspace and if so, whether the crawlspace was flooded. To assess the relative distribution of flooded and dry crawlspaces across the country, the percentages of both were calculated per province. In addition, we visualized the proportion of crawl space flooding in relation to surface altitude, for which we used a Dutch elevation GIS layer from Algemeen Hoogtebestand Nederland (AHN)^90^. From the submissions from participants with a crawlspace, 57 and 62 mosquitoes were randomly selected for molecular identification from locations with dry crawlspaces and flooded crawlspaces, respectively. This subset of samples was used to assess the relationship between crawlspace status (dry vs. flooded) and *Cx. pipiens*s.s. biotype composition. Categorization and selection of the data points, as well as creation of all maps, were performed using QGIS software (Version 3.22 ‘Białowieża’^53^,) and Microsoft Excel. Administrative boundary data were obtained from Statistics Netherlands (CBS): ‘CBS Gebiedsindelingen 2016–2025’ (https://www.cbs.nl/nl-nl/dossier/nederland-regionaal/geografische-data/cbs-gebiedsindelingen).

### Species and biotype identification

Subsets of *Cx. pipiens* s.s.*/torrentium*, based on human population density levels or crawlspace status were selected. Subsequently, samples were identified to the species and biotype level following the molecular diagnostics assay developed by Vogels et al.^24^, with changes to the protocol as described earlier^22^. In short, DNA was extracted from mosquito legs by lysis in a 4 M ammonium hydroxide solution. Species and biotype identification was performed using a probe-based Real Time PCR protocol, using biotype *molestus* and biotype *pipiens* primer/probe sets targeting the CQ11 flanking region. Samples which tested positive for heterozygosity (i.e. positive for both probes) were classified as *pipiens/molestus* hybrids. Samples for which the analysis did not result in a positive signal for any of the *Cx. pipiens* s.s. probes were retested with a *Cx. torrentium*-specific primer- and probe combination. Material from adult lab-reared *Cx. pipiens* biotype *pipiens* and biotype *molestus* were used as positive controls for the biotype assay. Larval material of morphologically identified *Cx. torrentium* was used as positive control for the *Cx. torrentium* assay. All Real Time PCRs were performed on a CFX Opus qPCR machine (Bio-Rad Laboratories, CA, USA).

### Blood-meal identification

Blood-meal analyses were performed as described by Blom et al.^22^. In short, abdomens of blood-engorged *Cx. pipiens* s.s.*/torrentium* specimens were collected and homogenized, followed by DNA extraction using the DNEasy Blood & Tissue kit protocol (Qiagen, Hilden, Germany). DNA isolates were amplified using primers targeting the CytB region and, in case of initial unsuccessful amplification, primers targeting the 16S region^91–93^. All PCR reactions were performed on a T100 Thermal Cycler (Bio-Rad Laboratories, CA, USA). PCR products were first visualized on gel (1% agarose, 4 µl Midori Green (NIPPON genetics EUROPE, GmbH, Düren, Germany)), and successful amplicons were subsequently submitted for Sanger sequencing as described earlier^22^. Acquired sequences were trimmed, and host origin was identified using NCBI BLAST with a pairwise identity threshold of ≥ 95%.

### Statistical analysis

The proportions of biotype *pipiens*, biotype *molestus* and *pipiens/molestus* hybrids between human population density levels was tested using Pearson’s chi-square test. Additionally, the proportion of biotype *pipiens*, *molestus* and *pipiens/molestus* hybrids submitted from locations with dry or wet crawl spaces was tested using Pearson’s chi-square test. In both cases, post-hoc pairwise comparisons of the biotype composition between the different categories were performed with Bonferroni correction, to reduce the probability of type 1 error. Additionally, to assess the combined effects of human population density and crawlspace flooding on *Cx. pipiens* s.s. biotype composition, a multinomial logistic regression model was performed. To this end, we used a combined subset of samples for which both variables (crawlspace status and human population density) were determined by integrating the subsets used for the human population density and crawlspace status analyses. For this analysis, human population density and crawlspace status were included as explanatory variables, with biotype composition as response variable. All statistical analyses were performed in RStudio 2023.06.0^94^.

## Electronic supplementary material

Below is the link to the electronic supplementary material.

Supplementary Material 1

## Data Availability

Bloodmeal raw reads were submitted to the NCBI Biosample database under the accession numbers SAMN50589878 to SAMN50590084.
