# Supplementary material for: Biotype dynamics of the common house mosquito Culex pipiens in the anthropogenic environment in winter: a citizen science approach
Source: Sci Rep. 2026 Jun 13;16:31779. doi: 10.1038/s41598-026-55124-5 (PMC13654805; doi:10.1038/s41598-026-55124-5)
Supplement: Supplementary file 1 [file 41598_2026_55124_MOESM1_ESM.docx]

Supplementary material

**Table S1.** Overview of the questions and answer options included in the Muggenradar questionnaire.

| **Question** | **Answer options** |
| --- | --- |
| Do you have any pets at home, and if so, which ones? | Yes  Bird  Cat  Dog  Rabbit  Rodent  Other  No |
| Do you have a crawlspace underneath your residence and if so, is it filled with water? | Yes, and it is (partially) filled with stagnant water.  Yes, and it is dry.  No |
| Did you experience any mosquito nuisance? | Yes, on (date)  No |
| Where did you collect the mosquito? | Bathroom  Bedroom  Kitchen  Living room  Other; |
| How many mosquitoes did you find indoors? | Between 1 – 5  Between 6 – 10  More than 10 |

**Table S2.** Number of georeferenced submissions (including filled-out questionnaire) to the Muggenradar platform between February 16^th^ and February 26^th^, 2021, divided per province. Per province, the number of submissions per inhabitant was calculated, based on the number of inhabitants as of September 2022 [1]. In addition, the total number and percentages of submissions with crawlspaces, both dry and wet, are displayed. Percentages of dry and wet crawlspaces are based on residences with crawlspaces only.

| **Province** | **Total** | **No. of inhabitants (Sep. 2022)** | **No. of submissions per inhabitant (* 10.000)** | **Residences with crawlspaces (%)** | **Dry crawlspace (%)** | **Wet crawlspace (%)** |
| --- | --- | --- | --- | --- | --- | --- |
| Groningen | 223 | 595,011 | 3.7 | 155 (69.5) | 120 (77.4) | 35 (22.6) |
| Friesland | 245 | 658,747 | 3.7 | 202 (82.4) | 160 (79.2) | 42 (20.8) |
| Drenthe | 219 | 501,327 | 4.4 | 167 (76.3) | 144 (86.2) | 23 (13.8) |
| Overijssel | 294 | 1,181,720 | 2.5 | 243 (82.7) | 217 (89.3) | 26 (10.7) |
| Flevoland | 100 | 441,874 | 2.3 | 91 (91.0) | 83 (91.2) | 8 (8.8) |
| Gelderland | 554 | 2127,493 | 2.6 | 400 (72.2) | 370 (92.5) | 30 (7.5) |
| Utrecht | 667 | 1,382,302 | 4.8 | 524 (78.6) | 456 (87.0) | 68 (13.0) |
| Noord-Holland | 1103 | 2,941,163 | 3.8 | 789 (71.5) | 661 (83.8) | 128 (16.2) |
| Zuid-Holland | 1067 | 3,787,452 | 2.8 | 844 (79.1) | 653 (77.4) | 191 (22.6) |
| Zeeland | 48 | 390,636 | 1.2 | 23 (47.9) | 16 (69.6) | 7 (30.4) |
| Noord-Brabant | 405 | 2,617,331 | 1.5 | 164 (40.4) | 145 (88.4) | 19 (11.6) |
| Limburg | 94 | 1,124,206 | 0.8 | 46 (48.9) | 44 (95.7) | 2 (4.3) |
| **The Netherlands** | **5019** | **17,749,262** | **2.8** | **3648 (72.7)** | **3069 (84.1)** | **579 (15.9)** |

**Figure S1.** Map depicting the proportion of Muggenradar submissions originating from residences without a crawlspace, with a dry crawlspace, and with a wet (flooded) crawlspace per province. A relatively high percentage of crawlspace flooding (20%) can be observed in the coastal provinces, compared to a lower percentage of crawlspace flooding (10.8%) in inland provinces. The map was created by the authors, using QGIS software (version 3.22 ‘Białowieża’; QGIS Development Team, 2021). Administrative boundary data were obtained from Statistics Netherlands (CBS): ‘CBS Gebiedsindelingen 2016-2025’ (https://www.cbs.nl/nl-nl/dossier/nederland-regionaal/geografische-data/cbs-gebiedsindelingen).

**Figure S2.** Map displaying the human population density per municipality, expressed in number of addresses per km^2^ [1]. The map was created by the authors, using QGIS software (version 3.22 ‘Białowieża’; QGIS Development Team, 2021). Administrative boundary data were obtained from Statistics Netherlands (CBS): ‘CBS Gebiedsindelingen 2016-2025’ (https://www.cbs.nl/nl-nl/dossier/nederland-regionaal/geografische-data/cbs-gebiedsindelingen).

**Table S3.** Results of the statistical analyses performed on the different population density levels used in our study: low (<500 postal codes/km^2^), low-medium (500-1000 postal codes/km^2^), medium (1000-1500 postal codes/ km^2^), medium-high (1500-2500 postal codes/km^2^), and high (>2500 postal codes/km^2^). Overall differences were calculated using Pearson’s chi-square test and pairwise comparisons were corrected with a Bonferroni correction. Adjusted p-values < 0.05 are depicted in bold.

| **Pairwise comparison** | | **Χ^2^-value** | **df** | **p-value** | **Adjusted p- value** |
| --- | --- | --- | --- | --- | --- |
| Pop. Density | |  |  |  |  |
| Low | Low-medium | 7.075 | 2 | 0.029 | 0.291 |
| Low | Medium | 10.12 | 2 | 0.006 | 0.063 |
| Low | Medium-high | 16.213 | 2 | < 0.001 | **0.003** |
| Low | High | 33.367 | 2 | < 0.001 | **< 0.001** |
| Low-medium | Medium | 6.2433 | 2 | 0.044 | 0.441 |
| Low-medium | Medium-high | 8.1749 | 2 | 0.017 | 0.168 |
| Low-medium | High | 13.478 | 2 | 0.001 | **0.012** |
| Medium | Medium-high | 1.0382 | 2 | 0.595 | 5.950 |
| Medium | High | 11.464 | 2 | < 0.001 | **0.032** |
| Medium-high | High | 6.1538 | 2 | 0.046 | 0.461 |
| Overall | | 44.324 | 8 | 4.94E-07 |  |

**Table S4.** Overview of the successful blood meal identifications originating from *Cx. pipiens* s.s. mosquitoes submitted to Muggenradar. Distinctions are made between biotype *molestus,* biotype *pipiens*, *pipiens/molestus* hybrids, and specimens of which the molecular identification was unsuccessful (NA).

| ***Culex pipiens* s.s. biotype** | **Blood meal host** | **No.** |
| --- | --- | --- |
| *Molestus* | *H. sapiens* | 168 |
|  | *N. hollandicus* | 1 |
|  | *C. lupus familiaris* | 2 |
| *Pipiens* | *C. palumbus* | 1 |
|  | *H. sapiens* | 2 |
| *pipiens/molestus* hybrid | *H. sapiens* | 22 |
| NA | *B. taurus* | 1 |
|  | *G. gallus* | 1 |
|  | *H. sapiens* | 9 |

References

[1] CBS. (2023). *Inwonertal per provincie, 1 september 2022*. https://www.cbs.nl/nl-nl/maatwerk/2023/07/inwonertal-per-provincie-1-september-2022
